# Aging and prostate health: meta-analytic insights into age-related prostatic disorders

**DOI:** 10.3389/fonc.2026.1744306

**Published:** 2026-04-10

**Authors:** Yuqian Cui, Hui Zhuo

**Affiliations:** 1 College of Medicine, Southwest Jiaotong University, Chengdu, Sichuan, China; 2 Department of Urology, The Affiliated Hospital of Southwest Jiaotong University, The Third People’s Hospital of Chengdu, Chengdu, Sichuan, China

**Keywords:** aging, benign prostatic hyperplasia, detection bias, meta-analysis, prostate cancer, prostate health, systematic review

## Abstract

**Background and purpose:**

Prostate health conditions, including benign prostatic hyperplasia (BPH) and prostate cancer (PCa), have historically been linked to aging. Nevertheless, previous studies have not sufficiently addressed detection bias arising from increased prostate screening in older males, which may have artificially inflated incidence rates. This systematic review and meta-analysis aimed to synthesize available evidence on the relationship between aging and prostate disorders while accounting for potential detection bias.

**Materials and methods:**

A systematic literature search was conducted across PubMed, Scopus, Web of Science, Google Scholar, and the NCBI Trials registry for studies published up to June 2024. Studies reporting age-related prevalence of risk estimates for PCa or BPH were included. Pooled prevalence estimates were synthesized using both fixed-effects and random-effects meta-analytic models. Between-study heterogeneity was assessed using Cochran’s Q test and the I^2^ statistic.

**Results:**

Eighteen studies (12 on PCa and six on BPH) involving 21483 participants were included in the final analysis. Under the random-effects model, the pooled prevalence estimate for PCa was 0.966 (95% CI: 0.886–0.991), while pooled prevalence estimate for BPH was 0.132 (95% CI: 0.068–0.241). substantial between-study heterogeneity was observed (I^2^ >95%). These pooled estimates represent summary prevalence proportions rather than comparative odds ratios and therefore reflect the burden of prostate disorders across aging male populations rather than causal risk effects.

**Conclusions:**

This meta-analysis demonstrates a substantial prevalence of prostate disorders in aging male populations. However, considerable heterogeneity across studies suggests that differences in population characteristics, study design, and screening intensity may influence the observed associations. These findings highlight the need for future epidemiological studies that better distinguish biological aging effects from diagnostic and surveillance-related factors.

**Systematic review registration:**

https://www.crd.york.ac.uk/prospero/, identifier CRD420251021675

## 1 Introduction

Aging is a complex biological process characterized by progressive declines in cellular function and homeostasis (1, 2). In this study, the term “aging” refers to chronological age as reported in epidemiological studies rather than molecular aging markers or genetic indicators of senescence. In men, advancing age is associated with an increased risk of prostate disorders, notably benign prostatic hyperplasia (BPH) and prostate cancer (PCa). As individuals age, cellular impairment, altered hormonal regulation, and diminished tissue repair capacity may contribute to the development of these prostate conditions (3). Epidemiological trends underscore this concern: after a two-decade decline, PCa incidence rates have been rising by approximately 3% annually since 2014 (4, 5). In contrast, overall cancer rates in men have shown more favorable trends than in women, highlighting distinct variations in cancer patterns across populations (5).

Prostate health is a major concern for aging men, particularly due to the high prevalence of BPH and PCa (6). While BPH is more common, PCa has received greater research attention due to its malignant nature and higher mortality risk (7, 8). Genetic studies reveal substantial differences between BPH and PCa, suggesting that BPH, despite arising from the same anatomical site, is more strongly influenced by aging and environmental factors rather than serving as a direct precursor to cancer. Differentiating between these conditions is crucial for effective management (9, 10).

The prevalence of BPH increases markedly with age, affecting approximately 50% of men aged 50–59 years and up to 90% of those aged 80 years and older (3). BPH-related lower urinary tract symptoms (e.g., frequent urination, urgency, nocturia) can substantially impair quality of life and impose a significant healthcare burden (11–13). As a chronic and widespread condition, BPH also carries a considerable financial burden. Meanwhile, PCa risk rises with age, with approximately one-third of cases occurring in men over 65 years. Biological aging processes –including cumulative oxidative stress, genomic instability, and DNA damage– likely contribute to carcinogenesis and progression of PCa, which may explain the higher likelihood of advanced disease in older patients (14, 15).

Given the rapid aging of the global population and recent changes in PCa incidence trends, clarifying the relationship between aging and prostate health is of paramount importance. However, findings from individual studies have been inconsistent, and several investigations suggest that increased prostate-specific antigen (PSA) screening among older men may introduce detection bias, potentially inflating the apparent incidence of prostate disorders in this population. Previous epidemiological analyses have highlighted how differential screening intensity across age groups may influence reported disease prevalence and risk estimates (16, 17).

Although numerous epidemiological studies have examined this relationship, their findings remain heterogeneous. Differences in study design, screening intensity, population characteristics, and diagnostic practices may partly explain these discrepancies. In particular, increased screening frequency among older men may lead to earlier or more frequent detection of asymptomatic disease, thereby exaggerating the apparent association between age and prostate disorders.

The aim of this systematic review and meta-analysis was to quantitatively evaluate the association between chronological age and the risk of PCa and BPH while considering the potential influence of detection bias related to differential screening practices across age groups. Unlike previous studies that have examined PCa or BPH separately, the present meta-analysis integrates evidence across both malignant and benign prostate conditions within a single analytical framework, allowing a comparative assessment of age-related patterns across distinct prostate disease entities.

## 2 Methods

This systematic review and meta-analysis was retrospectively registered in the International Prospective Register of Systematic Reviews database (PROSPERO number: CRD420251021675), and conducted in accordance with the guidelines outlined in the Preferred Reporting Items for Systematic Reviews and Meta-analyses (PRISMA) 2020 statement.

### 2.1 Search strategy

Each step of the review process was conducted independently by two authors. Disagreements regarding study eligibility were resolved through discussion with a third senior researcher who was not involved in writing the article. The research question was formulated using the Population, Intervention, Control, Outcome, and Study design (PICOS) framework.

A comprehensive literature search was performed on July 17, 2024, using the databases MEDLINE (via PubMed), Scopus, Web of Science Core Collection, Google Scholar, and the National Center for Biotechnology Information (NCBI) Trials registry. Gene expression dataset-based studies were also searched, and the references of retrieved articles were manually screened to identify any additional sources.

The search strategy used a combination of Medical Subject Heading (MeSH) terms and free-text keywords related to prostate, PCa, BPH, and aging. The general search string was: (“aging” [free-text] OR “age” [free-text] OR “older men” [free-text] OR “elderly” [free-text] OR “aged” [MeSH] OR “senescence” [free-text]) AND (“prostate cancer” [free-text] OR “prostatic neoplasms” [MeSH] OR “prostate malignancy” [free-text] OR “prostate carcinoma” [free-text]) AND (“benign prostatic hyperplasia” [MeSH] OR “BPH” [free-text] OR “benign prostatic enlargement” [free-text] OR “benign prostatic disease” [free-text]) AND (“risk” [free-text] OR “risk factor*” [free-text] OR “incidence” [free-text] OR “prevalence” [free-text] OR “development” [free-text] OR “association” [free-text] OR “odds” [free-text] OR “hazard” [free-text]). Database-specific search adjustments and the full search strategy for each source are provided in **Supplementary Data Sheet 1**.

### 2.2 Selection criteria

Using the PICOS framework, the study eligibility criteria were defined as follows.

Population (P): general male populations.

Intervention/Exposure (I): chronological aging, defined as increasing chronological age assessed either as a continuous variable or through comparisons between predefined age categories (commonly ≥50 or ≥60 years versus younger reference groups) in the original studies. In the context of this meta-analysis, “aging” refers specifically to chronological age rather than molecular or genetic aging biomarkers).

Comparison (C): not applicable; studies comparing different age groups or assessing age as a continuous variable were included.

Outcome (O): development (incidence) or risk of BPH and/or PCa.

Stude design (S): observational or (non-)randomized trials reporting relevant outcomes.

Studies were included if they met the following criteria:(i) reported on the overall incidence, prevalence, or risk of PCa and/or BPH in the general male population; (ii) utilized an eligible study design (including observational, cohort, case-control, cross-sectional studies, or randomized/non-randomized controlled trials); (iii) were available in any language and any publication status (published, in-press, or grey literature). Studies were excluded if they (i) were restricted to highly specific subpopulations (e.g., men with prior prostate surgery, occupational cohorts, or unique genetic groups) or focused only on particular subtypes of PCa or BPH rather than overall disease incidence; (ii) provided only descriptive or qualitative findings without statistical analysis; (iii) were non-original publications, such as review articles, systematic reviews, meta-analyses, case reports, editorials, commentaries, conference abstracts without sufficient data, or letters to the editor.

### 2.3 Data extraction

For each included study, key characteristics and outcome data were extracted. This included the author names, publication year, study location, and sample size. Reported measures of association (e.g., odds ratios [ORs], relative risks or prevalence ratios) describing the relationship between chronological age and PCa or BPH were recorded when available. In most studies, age was analyzed either as a continuous variable or as categorical comparisons between older and younger populations (most commonly ≥50 years versus <50 years or decade-based age strata).

### 2.4 Quality assessment and data synthesis

The quality of the included studies were assessed using the Newcastle-Ottawa Scale (**Supplementary Data Sheet 2**) for observational studies (18). To evaluate the robustness of the results and the influence of study quality, sensitivity analyses were performed. Publication bias was assessed using funnel plots and Egger’s regression test (19). Funnel plots were used to visually assess asymmetry in the distribution of effect sizes across studies. Egger’s regression test was conducted by regressing precision-weighted effect sizes against their standard errors. However, in meta-analyses of prevalence estimates, funnel plot asymmetry and Egger’s test may reflect small-study effects or methodological heterogeneity rather than true publication bias. Therefore, these analyses were interpreted cautiously and primarily used as exploratory tools to evaluate potential small-study influences on the pooled estimates. A statistically significant *p*-value suggested possible publication bias and potential distortion of the pooled estimates.

### 2.5 Statistical analysis

All statistical analyses were performed using R software (version 4.3.1; R Foundation for Statistical Computing).

Because several included studies reported event counts and sample sizes rather than direct comparative risk measures, pooled prevalence estimates were synthesized using generalized linear mixed models. In this framework, the binomial distribution was assumed for the event counts, and a logit link function was applied to model the prevalence proportions. The logit transformation allows appropriate modeling of proportions bounded between 0 and 1 while stabilizing variance and accounting for study-level heterogeneity. Random intercepts were incorporated to capture between-study variability in prevalence estimates.

Both random-effects and fixed-effects models were fitted to obtain pooled prevalence estimates with corresponding 95% confidence intervals (CIs). Given the expected clinical and methodological diversity across studies, the random-effects model was considered the primary analytical framework, while the fixed-effects model was presented for comparison.

Between-study heterogeneity was assessed using Cochran’s Q statistic, tau-squared (τ²), and the I² statistic. I² values of 25%, 50%, and 75% were interpreted as low, moderate, and high heterogeneity, respectively. Because substantial heterogeneity was anticipated, pooled estimates were interpreted cautiously as descriptive summary measures rather than precise universal prevalence values. Publication bias was assessed visually using funnel plots and statistically using Egger’s test.

## 3 Results

### 3.1 Study characteristics

**Figure 1:**
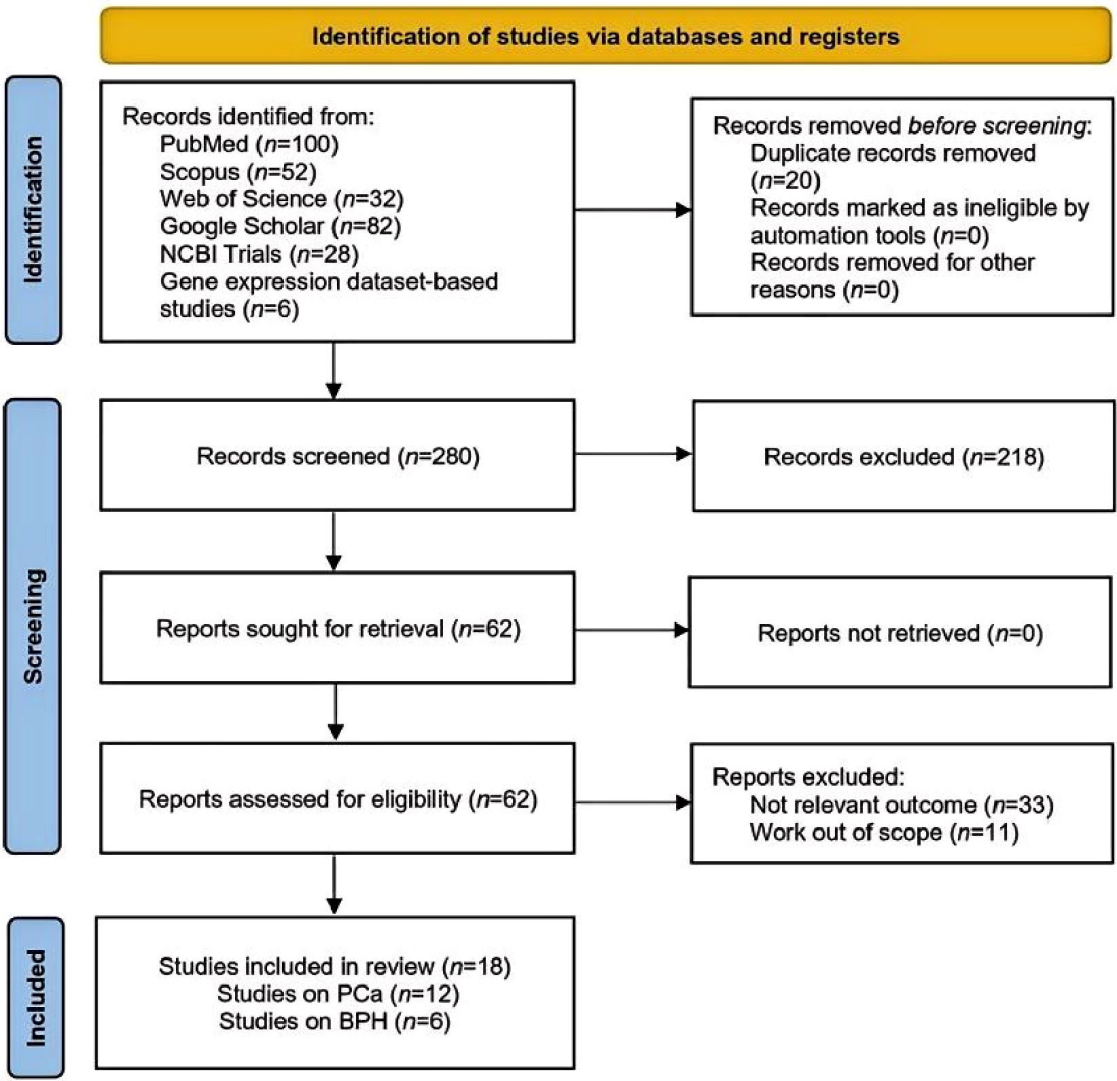
The PRISMA flow diagram illustrates the process of searching and selecting literature for studies and trials included in the analysis.

A total of 300 articles were initially identified for inclusion; 100 from PubMed, 52 from Scopus, 32 from Web of Science, 82 from Google Scholar, 28 from NCBI Trials, and six from gene expression dataset-based studies. After removing 20 duplicate records, 218 more were excluded based on title and abstract screening. Out of the 62 full-text articles assessed (30 on BPH, 32 on PCa), 44 were excluded due to lack of age-stratified outcome data (*n* = 17), absence of extractable quantitative estimates (*n* = 11), focus on highly specific clinical subpopulations (*n* = 9), and outcomes unrelated to overall prostate disease risk (*n* = 7). All of the eighteen studies (12 on PCa and six on BPH) were included through PubMed (20–37). The study selection process followed PRISMA 2020 guidelines, and the identification, screening, eligibility, and inclusion of studies are summarized in a PRISMA flow diagram (**Figure 1**).

Across the included studies, 12 PCa cohorts comprising 3,483 participants reported prevalence estimates ranging from 69% to 100%. Six BPH studies, involving more than 18,000 participants, reported prevalence estimates ranging from 4% to 28%. Although these data suggest increasing disease occurrence with advancing age, variability in diagnostic practices and case definitions across geographic settings likely influenced prevalence estimates. **Table 1** summarizes the key characteristics of the included studies.

**Table 1:** Baseline characteristics of the included studies.

Author (year)	Country	Sample size	Disease	Prevalence (%)	NOS score
Gerhauser et al., 2018 (20)	Germany	324	PCa	73	9
Grasso et al., 2012 (21)	USA	121	PCa	98	9
Abida et al., 2019 (22)	USA	444	PCa	93	9
Robinson et al., 2016 (23)	USA	150	PCa	98	9
Stotsack et al., 2020 (24)	USA	424	PCa	100	9
Baca et al., 2013 (25)	USA	82	PCa	69	7
Barbieri et al., 2012 (26)	USA	123	PCa	91	7
Fraser et al., 2017 (27)	Canada	477	PCa	96	9
Kumar et al., 2016 (28)	USA	176	PCa	35	6
Hieronymus et al., 2014 (29)	USA	104	PCa	97	9
Rang et al., 2018 (30)	China	65	PCa	100	9
Abeshouse et al., 2015 (31)	USA	333	PCa	98	9
Xiong et al., 2020 (32)	China	8563	BPH	22.7	9
Parsons et al., 2006 (33)	USA	422	BPH	21	9
Meigs et al., 2001 (34)	USA	1709	BPH	19.4	7
Kok et al., 2009 (35)	The Netherlands	4353	BPH	4.13	9
Wu et al., 2020 (36)	China	795	BPH	27.2	9
Pan et al., 2014 (37)	China	1052	BPH	24	9

BPH, Benign prostatic hyperplasia; NOS, Newcastle-Ottowas scale; Pca, Prostate cancer.

### 3.2 Random-effects model

Because substantial heterogeneity was observed across studies, the random-effects model was considered the primary analysis. Across the 12 PCa studies including 2,823 participants, the pooled prevalence estimate was 0.966 (95% CI: 0.886–0.991). This estimate represents the proportions of PCa cases observed within the analyzed populations rather than a comparative OR between age groups. Heterogeneity was substantial (τ²=4.656; Q = 351.42, df=11, *p* < 0.01; I²=97%).

For BPH, six studies involving 16,894 participants yielded a pooled prevalence estimate of 0.132 (95% CI: 0.068–0.241), again with very high heterogeneity (τ²=0.839; Q = 736.2, df=5, *p* < 0.01; I²=99%). As with PCa, these values reflect pooled prevalence proportions derived from event counts reported in the included studies. Despite inter-study variability, all studies consistently indicated a higher prevalence of BPH with increasing age.

**Figure 2:**
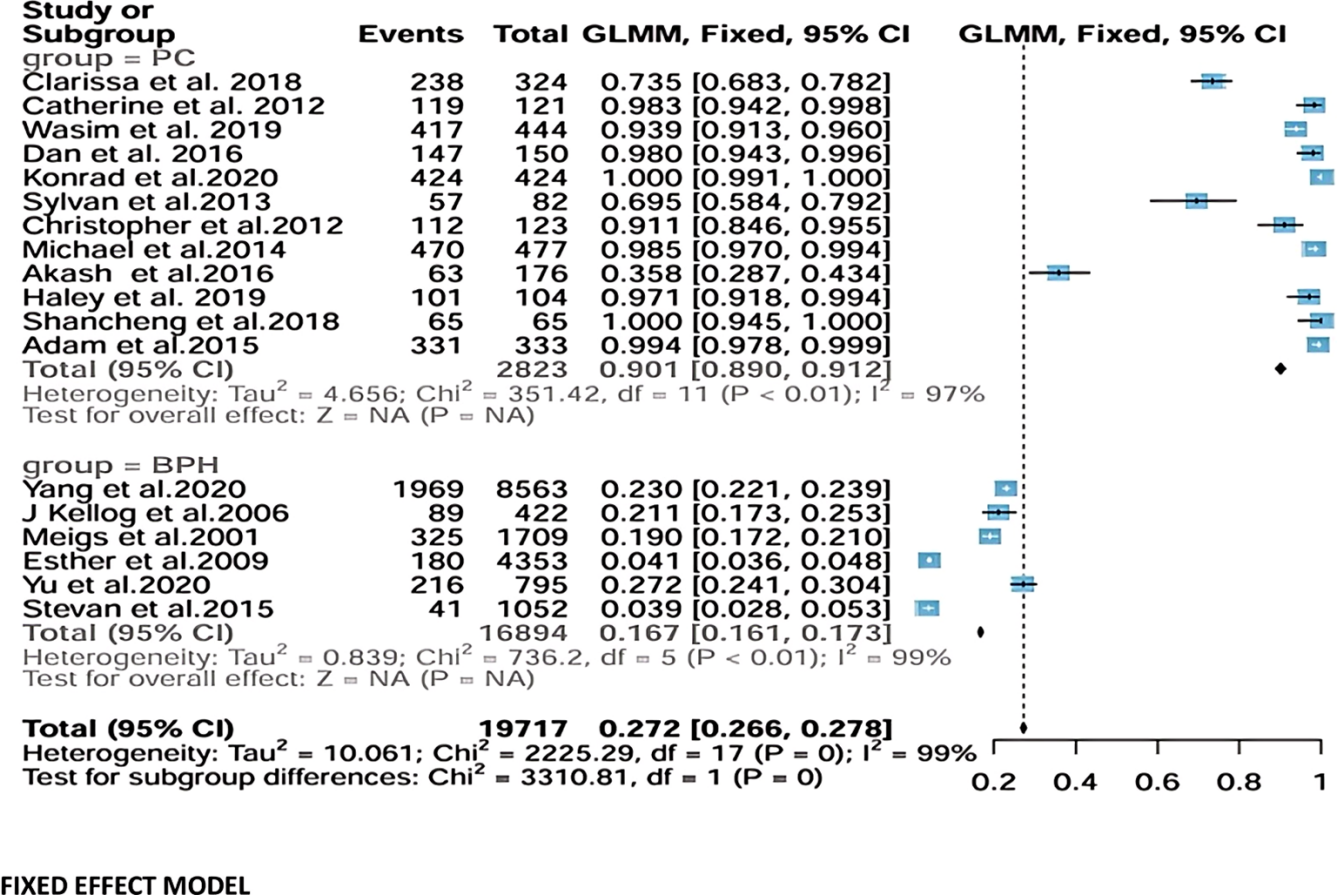
Random-effects model analysis.

When all 18 studies (19,717 participants) were analyzed together, the overall pooled prevalence estimate for prostate disorders was 0.838 (95% CI: 0.535–0.959). However, extremely high between-study heterogeneity was observed (τ²=10.061; Q = 2225.29, df=17, *p* < 0.01; I²=99%). A test for subgroup differences indicated that age-related pattern was significantly stronger for BPH than for PCa (Q = 47.03, df=1, *p* < 0.01) (**Figure 2**).

The high heterogeneity observed across studies likely reflects differences in study design, population characteristics, diagnostic criteria, and screening practices across geographic regions. Consequently, pooled estimates should be interpreted as descriptive summaries of disease prevalence across heterogeneous study populations rather than precise estimates of age-related risk.

### 3.3 Fixed-effects model

Using a fixed-effects model, the pooled prevalence estimate for PCa was 0.901 (95% CI: 0.890–0.912), based on 12 studies with 2,832 participants. As in the random-effects analysis, this value represents the overall proportion of PCa cases observed within the analyzed study populations rather than a comparative OR between age groups. Heterogeneity remained high (τ²=4.656; Q = 351.42, df=11, *p* < 0.01; I²=97%).

For BPH, the pooled prevalence estimate was 0.167 (95% CI: 0.161–0.173) across six studies including 16,894 participants. Again, heterogeneity remained very high (τ²=0.839; Q = 736.2, df=5, *p* < 0.01; I²=99%).

**Figure 3:**
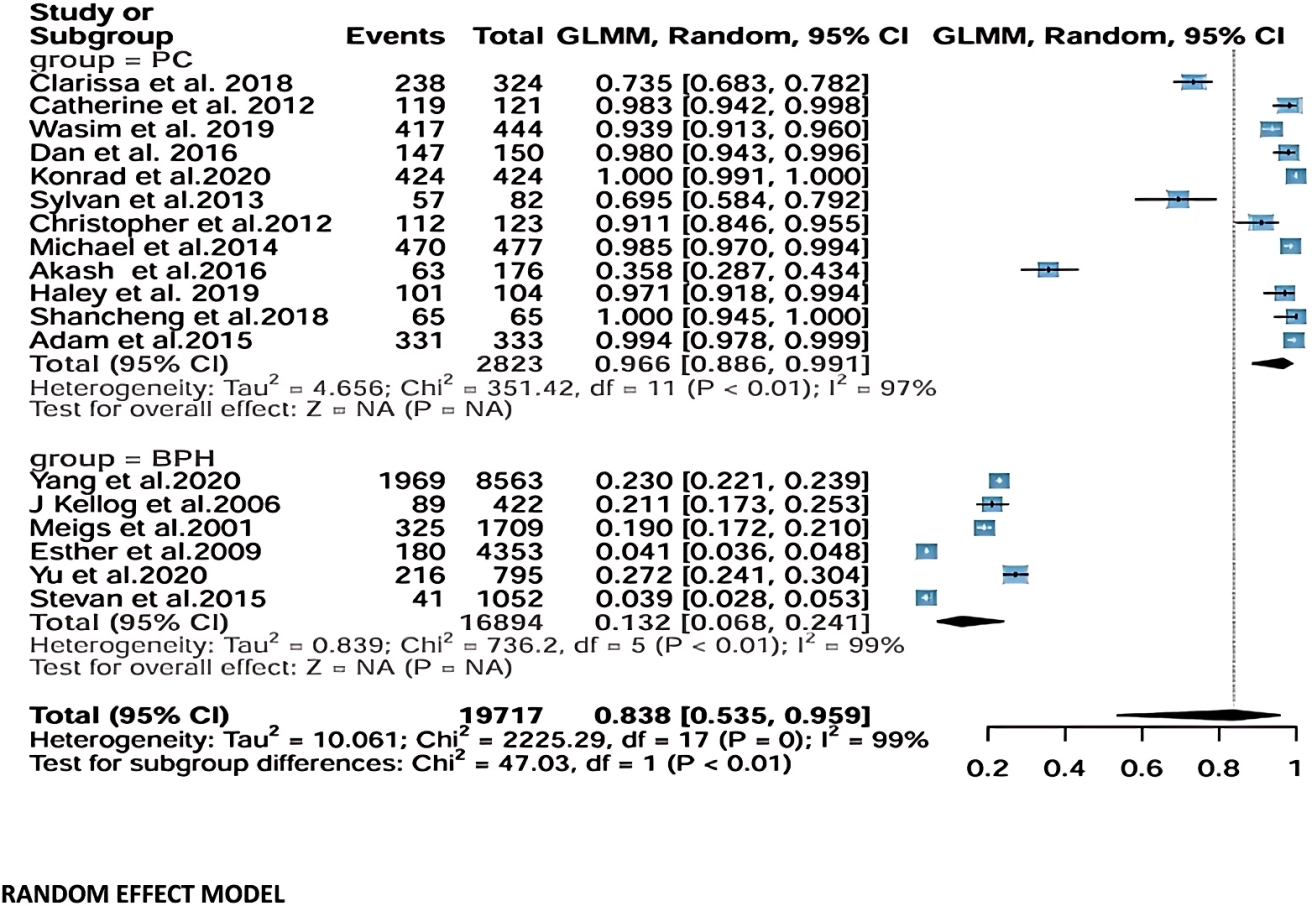
Fixed-effects model analysis.

When both outcomes were combined, the overall pooled prevalence estimate was 0.272 (95% CI: 0.266–0.278) across all 18 studies (19,717 participants), with extremely high heterogeneity (τ²=10.061; Q = 2225.29, df=17, *p* < 0.01; I²=99%) (**Figure 3**).

### 3.4 Subgroup analysis

The subgroup analysis revealed distinct patterns in the prevalence of prostate disorders when PCa and BPH studies were analyzed separately.

#### 3.4.1 PCa group

Across PCa studies, the pooled prevalence effect estimate was 0.901 (95% CI: 0.890–0.912). These estimates reflect the proportion of PCa cases reported within the analyzed study populations. Heterogeneity remained substantial (τ²=4.656; Q = 351.42, df=11, *p* < 0.01; I²=97%).

#### 3.4.2 BPH group

In the BPH subgroup, the pooled prevalence estimate was 0.167 (95% CI: 0.161–0.173). Heterogeneity remained very high (τ²=0.839; Q = 736.2, df=5, *p* < 0.01; I²=99%), reflecting methodological and biological variability among studies.

### 3.5 Publication bias analysis

**Figure 4:**
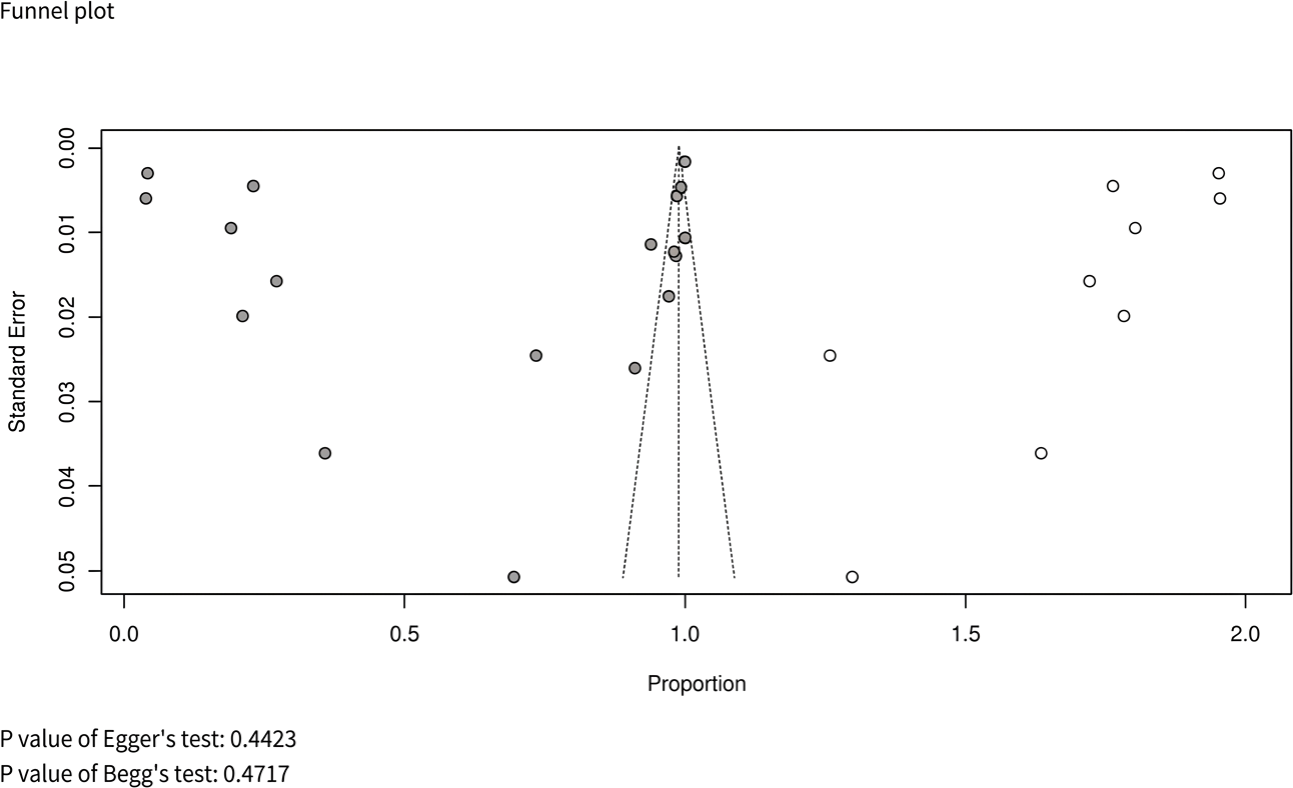
Funnel plot for publication bias assessment.

Egger’s test yielded a *p*-value of 0.442, suggesting no statistically significant evidence of small-study effects. Visual inspection of funnel plots (**Figure 4**) showed no substantial asymmetry. However, it should be noted that in meta-analyses of prevalence data, funnel plot asymmetry may arise from methodological heterogeneity or differences in study size rather than true publication bias. Therefore, these findings should be interpreted cautiously.

## 4 Discussion

This meta-analysis systematically investigated the relationship between aging and the prevalence of PCa and BPH, employing both random-effects and fixed-effects models to ensure a rigorous evaluation. The fixed-effects model summarized prevalence estimates across the included studies, while the random-effects model –designed to account for between-study variability– enabled us a more conservative assessment under substantial heterogeneity. The present study expands current knowledge by quantitatively synthesizing evidence on the relationship between chronological aging and two major prostate conditions within a single meta-analytic framework. While previous studies have often examined PCa or BPH separately, this analysis allows a direct comparison of age-related patterns across both diseases, providing broader insight into prostate health during aging. By synthesizing evidence from both epidemiological cohorts and molecularly characterized PCa datasets, this study provides a broader perspective on prostate health across the aging spectrum, bridging population-level disease burden with biological insights from tumor-focused research.

The pooled estimates derived from both random-effects and fixed-effects models represent prevalence proportions rather than classical ORs comparing age groups. Consequently, values below unity should not be interpreted as indicating a protective effect of aging. Instead, these estimates summarize the proportion of prostate disease cases observed within the analyzed study populations. In the present analysis, pooled prevalence estimates were 0.901 for PCa and 0.167 for BPH under the fixed-effects model, and 0.966 and 0.132, respectively, under the random-effects model. These findings highlight the substantial burden of prostate disorders observed among aging male populations.

The extremely high heterogeneity observed across studies (PCa I^2^ = 97%; BPH I^2^ = 99%) likely reflects differences in study design, population characteristics, diagnostic criteria, screening practices, and geographic settings. Such heterogeneity is common in meta-analyses that combine data from diverse populations and study types. Consequently, pooled estimates should be interpreted cautiously as descriptive summaries of disease prevalence rather than precise universal estimates of age-related disease risk. Nevertheless, the consistently high prevalence of prostate disorders observed across the included studies supports the well-established epidemiological observation that these conditions become increasingly common with advancing age.

Prostatic aging is characterized by cumulative oxidative DNA damage, telomere shortening, and genomic instability, which collectively contribute to carcinogenesis. Hormonal alterations —including declining testosterone levels and a relative increase in estrogen activity— may promote stromal proliferation and glandular hyperplasia, leading to BPH. Chronic inflammation and age-related changes in stromal-epithelial interactions may further reinforce a pro-proliferative and tumor-prone microenvironment. These molecular and tissue-level processes provide a biological rationale for the epidemiologic patterns observed in aging male populations.

The clinical and public health implications of these findings are substantial. Recognizing advancing age as a major factor associated with prostate disorders may encourage clinicians to prioritize early detection and appropriate management strategies, particularly in older populations. Early identification of PCa can improve clinical outcomes through timely treatment, while early diagnosis and management of BPH may substantially improve quality of life for affected individuals.

From a broader public health perspective, these findings support the development of targeted programs aimed at increasing prostate health awareness (38). Initiatives that promote appropriate screening practices, encourage healthy aging behaviors, and educate the public about early symptoms of prostate disease may contribute to reducing the overall disease burden and improving population health outcomes.

This work fills an important gap in the literature by synthetizing evidence on both BPH and PCa across broad age strata (39). In contrast to earlier studies that typically focused on a single prostate condition, the present meta-analysis provides a more comprehensive overview of prostate health across the aging spectrum. By incorporating evidence from multiple databases and diverse study designs, the analysis offers a broader synthesis of available evidence and highlights the complexity of age-related prostate disease.

Despite the strength of this approach, several important limitations should be acknowledged. First, the included studies varied considerably in their adjustment for potential confounding factors. Variables such as metabolic comorbidities, obesity, smoking status, hormonal profiles, lifestyle behaviors, environmental exposures, and genetic susceptibility may substantially influence prostate disease prevalence. Because these factors were inconsistently reported across the included studies, it was not possible to perform a pooled adjustment for these confounders in the present analysis. Consequently, the observed associations between chronological age and prostate disease prevalence should be interpreted as descriptive epidemiological patterns rather than evidence of a causal relationship. Second, many studies lacked long-term follow-up, limiting the ability to assess the sustained influence of aging on prostate disease occurrence. Third, inconsistent adjustment for confounders such as obesity, metabolic syndrome, smoking status, hormonal factors, environmental exposures, and other lifestyle factors may have contributed to variability in the reported prevalence estimates. Fourth, several included studies were observational and retrospective in design, making them more susceptible to selection bias and measurement error. Fifth, although Egger’s test did not detect significant publication bias (*p* = 0.44), selective reporting of positive findings cannot be completely excluded. Sixth, small sample sizes in some subgroups may have limited statistical power to detect more subtle associations. Finally, the review process was conducted by two authors, and although all steps were performed independently and systematically, the absence of an additional reviewer may have limited further cross-validation during study selection and data extraction.

Overall, this meta-analysis highlights the substantial burden of prostate disorders among aging male populations while emphasizing the complexity of interpreting epidemiological patterns across heterogenous studies. Future large-scale, longitudinal studies with standardized diagnostic criteria and comprehensive adjustment for potential confounders are needed to better clarify the relationship between aging and prostate disease and to improve strategies for prevention, early detection, and clinical management.

## 5 Conclusion

This meta-analysis highlights a substantial prevalence of PCa and BPH among aging male populations; however, due to heterogeneity and potential confounding factors, these findings should be interpreted as descriptive epidemiological summaries rather than causal estimates of age-related disease risk. The findings underscore the importance of early identification and appropriate management of prostate conditions, which may help reduce disease-related complications and improve patient outcomes. However, the substantial variability observed in PCa-related outcomes suggests that additional factors–such as genetic predisposition, lifestyle influences, and comorbidities–may contribute to the observed age-related patterns of prostate disease. A deeper understanding of these factors is essential for refining disease assessment strategies and developing targeted prevention approaches. Future research should prioritize methodological standardization to enhance the reliability and comparability of findings, ultimately leading to more precise evaluations of prostate disease patterns across aging populations.

## Data Availability

The original contributions presented in the study are included in the article/**Supplementary Material**. Further inquiries can be directed to the corresponding author.
