# Supplementary material for: Aging and prostate health: meta-analytic insights into age-related prostatic disorders
Source: Front Oncol. 2026 Apr 10;16:1744306. doi: 10.3389/fonc.2026.1744306 (PMC13106090; doi:10.3389/fonc.2026.1744306)
Supplement: Supplementary file 1 [file DataSheet1.docx]

**SEARCH STRATEGY**

**General search strategy:**

("aging" OR "age" OR "older men" OR "elderly" OR "aged" OR "senescence")

AND

("prostate cancer" OR "prostatic neoplasms" OR "prostate malignancy" OR "prostate carcinoma")

AND

("benign prostatic hyperplasia" OR "BPH" OR "benign prostatic enlargement" OR "benign prostatic disease")

AND

("risk" OR "risk factor*" OR "incidence" OR "prevalence" OR "development" OR "association" OR "odds" OR "hazard")

**Database-Specific Adjustments:**

***PubMed:***

("Aging"[Mesh] OR "Age Factors"[Mesh] OR aging[tiab] OR age[tiab] OR "older men"[tiab] OR elderly[tiab] OR aged[tiab] OR senescence[tiab])

AND

("Prostatic Neoplasms"[Mesh] OR "prostate cancer"[tiab] OR "prostatic neoplasms"[tiab] OR "prostate malignancy"[tiab] OR "prostate carcinoma"[tiab])

AND

("Prostatic Hyperplasia"[Mesh] OR "benign prostatic hyperplasia"[tiab] OR BPH[tiab] OR "benign prostatic enlargement"[tiab] OR "benign prostatic disease"[tiab])

AND

("Risk"[Mesh] OR "Risk Factors"[Mesh] OR risk[tiab] OR "risk factor*"[tiab] OR incidence[tiab] OR prevalence[tiab] OR development[tiab] OR association[tiab] OR odds[tiab] OR hazard[tiab])

***Scopus & Web of Science:***

(TITLE-ABS-KEY(aging OR age OR "older men" OR elderly OR aged OR senescence))

AND

(TITLE-ABS-KEY("prostate cancer" OR "prostatic neoplasms" OR "prostate malignancy" OR "prostate carcinoma"))

AND

(TITLE-ABS-KEY("benign prostatic hyperplasia" OR BPH OR "benign prostatic enlargement" OR "benign prostatic disease"))

AND

(TITLE-ABS-KEY(risk OR "risk factor*" OR incidence OR prevalence OR development OR association OR odds OR hazard))

***Google Scholar:***

("aging" OR "age" OR "older men" OR "elderly" OR "aged" OR "senescence")

AND

("prostate cancer" OR "prostatic neoplasms" OR "prostate malignancy" OR "prostate carcinoma")

AND

("benign prostatic hyperplasia" OR "BPH" OR "benign prostatic enlargement" OR "benign prostatic disease")

AND

("risk" OR "risk factors" OR "incidence" OR "prevalence" OR "development" OR "association" OR "odds" OR "hazard")
