# Supplementary material for: Aging and prostate health: meta-analytic insights into age-related prostatic disorders
Source: Front Oncol. 2026 Apr 10;16:1744306. doi: 10.3389/fonc.2026.1744306 (PMC13106090; doi:10.3389/fonc.2026.1744306)
Supplement: Supplementary file 2 [file DataSheet2.docx]

**Newcastle-Ottawa Scale analysis:**

**Manuscript title:** Aging and prostate health: Evidence-Based insights from a meta-analytic investigation

**NOS Domain and Criteria:**

| **Domain** | **Criteria** | **Stars** |
| --- | --- | --- |
| **Selection** | **1. Random sequence generation (true RCT)** | **★** |
|  | **2. Allocation concealment** | **★** |
|  | **3. Representative sample of elderly population** | **★** |
|  | **4. Clear description of exposure (aging factor clearly documented)** | **★** |
| **Comparability** | **5. Groups comparable at baseline (e.g., age, BMI)** | **★** |
|  | **6. Adjusted for potential confounders (e.g., socioeconomic, nutrition)** | **★** |
| **Outcome** | **7. Objective measurement of prostate health conditions (hospital records, clinical diagnoses)** | **★** |
|  | **8. Adequate follow-up period** | **★** |
|  | **9. Low attrition with intent-to-treat analysis or complete outcome reporting** | **★** |

**Criteria for Selection:**

- **Representativeness of the exposed cohort:** Inclusion of a truly representative elderly cohort.
- **Selection of non-exposed cohort:** Appropriate and comparable younger or control group.
- **Ascertainment of exposure:** Objectively documented, validated methods to confirm aging-related factors.
- **Demonstration that outcome was not present at start:** Documented clear evidence that prostate health conditions (BPH, PC) were not present at the outset.

**Quality Rating:**

- **High Quality:** 8-9 stars
- **Moderate Quality:** 5-7 stars
- **Low Quality:** ≤4 stars

**Newcastle-Ottawa Scale (NOS) Quality Assessment of Included Studies (Aging and Prostate Health)**

| **Study (PMID)** | **Author** | **Year** | **Selection (4★)** | **Comparability (2★)** | **Outcome (3★)** | **Total Score (9★)** | **Quality Rating** |
| --- | --- | --- | --- | --- | --- | --- | --- |
| 30537516 | Gerhauser | 2018 | ★★★★ | ★★ | ★★★ | 9 | High |
| 22722839 | Grasso | 2012 | ★★★★ | ★★ | ★★★ | 9 | High |
| 31061129 | Abida | 2019 | ★★★★ | ★★ | ★★★ | 9 | High |
| 26000489 | Robinson | 2016 | ★★★★ | ★★ | ★★★ | 9 | High |
| 32220891 | Stotsack | 2020 | ★★★★ | ★★ | ★★★ | 9 | High |
| 23622249 | Baca | 2013 | ★★★ | ★★ | ★★ | 7 | Moderate |
| 22610119 | Christopher | 2012 | ★★★ | ★★ | ★★ | 7 | Moderate |
| 28068672 | Fraser | 2017 | ★★★★ | ★★ | ★★★ | 9 | High |
| 26928463 | Kumar | 2016 | ★★★ | ★ | ★★ | 6 | Moderate |
| 25024180 | Hieronymus | 2014 | ★★★★ | ★★ | ★★★ | 9 | High |
| 28927585 | Rang | 2018 | ★★★★ | ★★ | ★★★ | 9 | High |
| 26544944 | Abeshouse | 2015 | ★★★★ | ★★ | ★★★ | 9 | High |
| 32583703 | Xiong | 2020 | ★★★★ | ★★ | ★★★ | 9 | High |
| 16608892 | Parsons | 2006 | ★★★★ | ★★ | ★★★ | 9 | High |
| 11520654 | Meigs | 2001 | ★★★ | ★★ | ★★ | 7 | Moderate |
| 19091352 | Kok | 2009 | ★★★★ | ★★ | ★★★ | 9 | High |
| 31058560 | Wu | 2020 | ★★★★ | ★★ | ★★★ | 9 | High |
| 26382806 | Kaplan | 2015 | ★★★★ | ★★ | ★★★ | 9 | High |
